# Youth post-discharge recovery and family adjustment after inpatient mental health treatment – study protocol for the longitudinal mixed-methods ReCoVer study

**DOI:** 10.3389/frcha.2026.1605414

**Published:** 2026-08-07

**Authors:** Amos-Silvio Erik Friedrich, Martina Zemp

**Affiliations:** Department of Clinical and Health Psychology, University of Vienna, Vienna, Austria

**Keywords:** adolescent, caregiver, child, family, inpatient treatment, mental health, needs, readmission

## Abstract

**Background:**

High readmission rates after child and adolescent inpatient treatment point to underrecognized issues in the post-discharge period. With a shift towards early reintegration in youth mental health care, the family environment youth return to is of particular importance for post-discharge outcomes, but there is currently a knowledge gap on family-level risk and protective factors in this context. Caregiver burden may act as a crucial mechanism linking family context to youth recovery.

**Methods and design:**

The ReCoVer study investigates caregivers’ perspectives on family care and youth mental health following inpatient psychiatric treatment (minimum 24-hour stay) of youth aged 7–21 in Germany, Austria, and Switzerland. In a mixed-methods design, ReCoVer combines a longitudinal panel survey with semi-structured interviews. Caregivers of youth who received inpatient mental health treatment (target *N* = 70) will be recruited during discharge planning or follow-up sessions within one month after discharge. At baseline (T0) and after three (T1) and six (T2) months, participants will report on youth outcomes, caregiver well-being, and family processes. Primary outcomes (inpatient readmission, mental health problems) will be analyzed with regard to the contribution of caregiver burden and other family factors. Interview and survey data will be synthesized to identify burden, resources, and needs of families around their child's return to home care.

**Discussion:**

Results from this study are expected to provide insights into family challenges and their associations with youth mental health in the immediate and medium-term post-discharge period.

**Clinical Trial Registration:**

https://clinicaltrials.gov/study/NCT06865573, NCT06865573.

## 1 Introduction

Child and adolescent mental health (CAMH) care is increasingly prioritizing least-intrusive treatment that is close to the community and family setting. As a result, inpatient treatment focuses on crisis stabilization and facilitating early transition to outpatient care, emphasizing the post-discharge period as essential for sustained improvements. However, high readmission rates within the first year after discharge point to deficiencies in the implementation of this step-down model (1). The transition out of hospital and return to home care must therefore be well-coordinated to ensure continued and adequate care (2). Models for improving post-discharge care generally address the central dimensions of family, school, community, and the healthcare system (3). Given the family's central role on its own and in relation to the other dimensions, this study focuses on the family environment to which children and adolescents return.

### 1.1 Post-discharge family context

Adequate follow-up care is critical for sustained recovery, yet youth are largely dependent on their family and caregivers to navigate healthcare services. Indeed, family characteristics predict youth service use to a significant extent (4), placing caregivers at the intersection of youth's clinical and caregiving context. Their perceptions, knowledge, and resources have been shown to be critical determinants of youth service use beyond objective need (5, 6). Despite this central role, family and caregiver factors are relatively understudied with regard to post-discharge service outcomes (7).

Beyond the role of caregivers in determining healthcare utilization, they often represent the primary social context youth recovery is situated in. Previous research has linked indicators of family functioning and support to improved outcomes during and after the transition out of hospital care (8–11). In a similar vein, qualitative studies focusing on the post-discharge context underscore the impact of the family environment in general, and caregivers in particular (12–14), a finding echoed by perspectives specific to the Austrian context (15). Research on adult patients has established family interactional processes as particularly prognostic, best evidenced from expressed emotion (EE) research showing that critical, hostile, or emotionally overinvolved family relations pose a transdiagnostic risk factor for adverse mental health trajectories (16, 17). For children and adolescents, parental critical attitudes towards their child's mental health problems have shown to be the most predictive aspect of EE, whereas other components such as emotional overinvolvement may not be adequately conceptualized for youth. The proposed mechanism is one of maintained or exacerbated symptom distress driven by the dysregulating effects of EE-associated behaviors. Across EE and related interactional conceptualizations, family members’ supportive reactions to youth problems (e.g., emotion-related parenting behaviors, dyadic coping) are regarded as a central aspect of a recovery-promoting family environment, whereas unsupportive parent-child interactions (e.g., criticism) may constitute important risk factors in this context (18, 19).

At the same time, parents and caregivers often report low competence and self-efficacy in caring for their child after discharge (20, 21), a pattern shown to be dynamically related to patient well-being in a German study on post-discharge reintegration (22). Youth problems requiring hospitalization can be a significant stressor for caregivers, almost all of whom report needing help at home (23). This strain is conceptualized as caregiver burden, defined as the objective demands of caring for individuals with (mental health) impairments and the associated subjective cognitive and emotional impact (24). While generally correlated with symptom severity across mental disorders (25), a recent meta-analytic review has concluded that caregiver burden is particularly high for caregivers of younger and hospitalized patients (26). Considerations of the family systems theory point out that mutual interconnections of family members and subsystems may result in reciprocal influence and reinforcement of distress within the systems (27, 28). This notion is supported by research demonstrating that parental distress impacts youth problems via dysfunctional processes and interactions in the parent-child relationship (29–31). Experiencing high burden in caring for youth with ongoing difficulties may thus both result in negative caregiver outcomes and impair their capacity to provide sensitive, recovery-promoting care to youth (25, 26).

For these reasons, a need for research on family risk and protective factors in transitional and post-discharge care has been identified (32, 33). For adult patients, caregiver support has been shown to improve long-term clinical and healthcare outcomes of transitional interventions (34). In CAMH, evidence is less conclusive as caregiver support has largely been “peripheralized” (6) in care services. Whereas initial stress of caregivers of youth undergoing inpatient psychiatric treatment may motivate higher involvement in treatment, sustained or chronic stress has been associated with adverse outcomes (8, 35). The direction of influence remains unclear, with one study suggesting that stress may be a direct contributor to, rather than only a correlate of, youth problems, as they did not in turn predict parenting stress over time (8). However, more recent findings partially support a mutual influence hypothesis postulating reciprocal associations between caregiver burden and youth mental health problems (36). Regardless of the specific direction, caregiver distress emerges as a potential key mechanism linking characteristics of the family context to youth mental health following their discharge from inpatient psychiatric care. Caregivers overwhelmed by managing ongoing difficulties and coordinating support systems may be less able to provide adequate support and more likely to seek out inpatient care services multiple times.

### 1.2 Research gaps

In summary, to ensure optimal outcomes, CAMH care should be *needs-based* and *family-focused*, i.e., tailored to the individual needs of young patients and their families (32). Therefore, assessing families’ burden and needs is a prerequisite to improving transitional and follow-up care after inpatient treatment. That said, there is a lack of knowledge about caregiver factors in this critical period, as they have rarely been studied specifically (23). Hence, research is needed to better understand how caregivers contribute, and can be supported to contribute, to youth mental health after their inpatient treatment (33, 37).

Literature on family factors in the post-discharge period is particularly sparse in the German-speaking countries. Studies from other regions and healthcare contexts, respectively, generally suggest the central role of caregiver factors on various intermediate processes and outcomes, e.g., access to follow-up care, parent-child relationships, and readmissions to inpatient care (1, 10, 38). A current review (7) generally supports the role of various family-level factors in CAMH readmissions but highlighted gaps concerning prospective studies on family processes (as opposed to, often only incidentally assessed, structural characteristics), particularly outside the US healthcare context. Given that healthcare systems substantially differ between countries and geographical regions, evidence from German-speaking regions is needed to bridge this gap. This is particularly relevant as inpatient treatment moves toward shorter lengths of stay, increasingly relying on post-discharge supports delivered in outpatient and community settings (39). The situation is especially pronounced in Austria, where inpatient capacity is comparably limited (40). In this context, caregiver and family factors play a central role in supporting continuity of care and post-discharge adjustment, but they also bear significant responsibility for managing and coordinating services. This may place considerable burden on them or exceed their available resources, resulting in suboptimal care for returning youth.

Interventions rarely explicitly target, engage, or involve caregivers despite calls for a family focus in post-discharge care (2). Likewise, care systems in the countries under study often fail to adequately incorporate family-focused care on a structural level (39, 41). In a German study focusing on CAMH care transitions, 35% of caregivers reported having missed consideration of the family system in discharge management and aftercare, wishing to have been more involved in specific interventions (42). It remains unclear how post-discharge family support services differ between German-speaking countries and whether recent developments have improved upon these shortcomings.

Methodologically, studies on caregivers of youth with mental health problems have often relied on general, unspecific measures of psychological distress. Specific measures to assess caregiver strain and burden, both in general and in relation to youth mental health, have been developed (43). These have proven to be valuable tools for examining how youth mental health problems affect their parents and other family members, and vice versa. Moreover, qualitative approaches have the potential to meaningfully expand on previous insights on caregiver perceptions (5). The present study thus examines caregiver burden and its role in the post-discharge period using context-specific measures and a mixed-methods approach.

## 2 Objectives, research questions, and hypotheses

The main goal of the ReCoVer study (German acronym: *Reintegration, Coping und Versorgung von Familien nach stationärer kinder- und jugendpsychiatrischer Behandlung* [English: Reintegration, coping, and care of families after inpatient child and adolescent psychiatric treatment]) is to examine caregivers’ perspectives of family care and youth mental health after their children's inpatient stay in a psychiatric institution in Germany, Austria, or Switzerland. The project aims to assess the needs of families in the context of their children's return to home care and to investigate the role of caregiver burden and family processes in the post-discharge period as key determinants of youth recovery and readmission, respectively.

### 2.1 Research questions (RQs)

RQ 1: Do family processes (i.e., caregiver burden, family functioning, parental psychopathology, family socioeconomic status) contribute to youth mental health outcomes after discharge from inpatient treatment?
RQ 1a: What family-level risk factors predict youth readmission to inpatient treatment?
RQ 1b: Do family processes predict youth mental health over time?
RQ 2: Is there a reciprocal relationship between youth mental health problems and caregiver burden over time?
RQ 3: Do parent-child interactions (i.e., relationship, dyadic coping, criticism) mediate the association between caregiver burden and youth mental health problems?
RQ 4: How do caregivers experience their children’s return to home care?
RQ 4a: What challenges and needs do caregivers report?
RQ 4b: What types of follow-up services do families receive and/or have access to?
RQ 4c: How do caregivers cope with ongoing difficulties in the post-discharge period?

### 2.2 Hypotheses

Our central hypothesis is that caregiver burden is a key mechanism for maintaining dysfunctional processes in affected families and, as a consequence, a determinant of youth mental health following discharge from psychiatric inpatient treatment. The following primary (confirmatory) hypotheses, corresponding to the main RQs outlined above, are derived from this main assumption. Specific hypotheses that will be tested beyond these will be preregistered individually before data analysis.

For RQ 1, we hypothesize that caregiver burden, along with indicators of family functioning, parental psychopathology, and socioeconomic status, predicts youth readmission to inpatient treatment and mental health problems over the study period beyond initial symptom severity. In RQ 2 and 3, we will examine reciprocal relationships between caregiver burden and youth mental health. We hypothesize that the two are related to each other both cross-sectionally and longitudinally (RQ 2), and that parent-child interaction mediates these associations (RQ 3). No specific hypotheses are related to RQ 4, as it is explorative in nature.

## 3 Methods and analysis

### 3.1 Open science practices and statement on generative AI

This study was registered at clinicaltrials.gov (unique identifier: NCT06865573). The current protocol serves to detail the rationale of the overall research project with its main research questions and hypotheses. Individual publications with specific research questions, hypotheses, and statistical analyses will be preregistered separately at the Open Science Framework prior to the respective data analysis, in accordance with the research questions specified here (see e.g., registration of preliminary analyses at https://osf.io/d34jv).

This protocol was reviewed against the SPIROS guidelines for reporting observational study protocols (44), which informed reporting where applicable. The completed checklist is provided in the supplementary materials. All additional supplementary materials for this protocol as well as anonymized data and analytical codes for future publications resulting from this project will be made available via the project repository at OSF (see https://osf.io/g54zq/).

In preparing this protocol, AI-based software was used to edit for brevity and clarity only. A declaration of the specific tools is included in the acknowledgements section.

### 3.2 Participants and eligibility criteria

The sample will consist of primary caregivers (parents or guardians identifying as responsible for caregiving regardless of the biological relationship to the child) of youth who received inpatient psychiatric treatment. Inclusion criteria are as follows: Caregivers are eligible for participation if they are (1) 18 years or older, (2) a primary caregiver of youth aged 7–21 years of any gender who received inpatient treatment in a psychiatric institution in Germany, Austria, or Switzerland (min. 24 h stay) within the past month (regardless of the diagnosis or the reason for the treatment, respectively), and (3) have lived in the same household with the patient since discharge. The patient age range reflects clinical practice in German-speaking countries, where transitional care policies extend specialized youth-focused care provision beyond the legal age of maturity at 18 years. Consequently, individuals aged 18–21 remain part of the relevant patient population treated at inpatient CAMH institutions across the German-speaking countries included in this study. Our inclusion criterion requiring a shared household further ensures that eligible young adults remain in comparable living situations to younger adolescents. Exclusion criteria are: (1) another caregiver of the same child has already participated in the study, (2) intellectual disability and (3) insufficient knowledge of German. Inclusion and exclusion criteria are screened via self-report before registration.

The target sample size comprises *N* = 70 caregivers. Within this sample, a subsample of up to *n* = 10 caregivers will be recruited for the additional interview module (see section 3.3.2) from participants indicating their interest at the end of the baseline survey. No purposive sampling for demographic or clinical variation is applied beyond balancing recruitment across the three countries. Recruitment will follow simple random sampling using a two-fold strategy: (1) open recruitment via online (social media) and offline (flyers, posters) advertisements, and (2) institutional recruitment initiated by staff at cooperating facilities during discharge planning or follow-up sessions.

Caregivers are compensated for their participation after the last time point. The amount is based on the extent of participation with a maximum amount of 40€ (10€ per completed time point plus an additional 5€ for completing the baseline survey and all surveys, respectively). Interview participation is further compensated with an additional 30€ upon completion of the interview.

### 3.3 Design

The mixed-methods study uses an embedded design consisting of a longitudinal survey module targeting all participants as well as an additional interview module targeting a subsample of interested participants.

#### 3.3.1 Survey module

Participants will complete online surveys consisting of items and questionnaires assessing sociodemographic information, clinical and treatment characteristics, experience with their child's return to home care, youth outcomes, caregiver well-being, and family processes (see section 3.5 for specific measures). This module consists of three time points: a baseline survey (T0: within the first month after the child's discharge) and two follow-up surveys (T1: three months after baseline; T2: six months after baseline). Completion time is estimated at up to 45 min (baseline) and up to 25 min (follow-up).

#### 3.3.2 Interview module

A subsample of *n* = 10 interested caregivers will be recruited for a semi-structured interview session to qualitatively explore their experience with their children's return to home care. To ensure broad reach, interviews will be conducted and recorded via the videoconferencing software Zoom, which has been shown to be practicable and viable in this context (45, 46).

### 3.4 Procedure

Interested participants are provided with a link to an online registration form, which will be used to screen for eligibility criteria, obtain informed consent, and collect e-mail addresses. Participant registration will be open from May 2025 through December 2026. Surveys are sent out via e-mail immediately after registration unless the child or adolescent is not yet discharged, in which case they are sent out one week after the scheduled date. Follow-up surveys are scheduled to be sent out automatically three (T1) and six months (T2) after completion of the baseline assessment. In addition to questionnaires, a list of relevant support services and emergency telephone numbers is displayed at the bottom of each assessment page given the anticipated high burden.

Opt-in for participation in the optional interview module is possible at the end of the baseline survey (T0). Informed consent is separately obtained for interview participation after explicitly informing about the processing and handling of interview data. Interviews will be conducted within three months, before the first follow-up assessment.

### 3.5 Measures

Table 1 provides an overview of the quantitative measures assessed at each time point. A complete list of self-developed items can be found in the project repository (https://osf.io/g54zq/).

**Table 1:** Overview of survey measures at each time point.

Measures	T0 (Baseline survey)	T1/T2 (Follow-up surveys)	Assessed constructs
Sociodemographic information	x	x (selected items, see section 3.5.1)	Age, gender, country of residence, language, education, income, financial stress, caregiving arrangement, migration background
Clinical and treatment characteristics	x	– (only upon readmission^a^)	Diagnosis, date of discharge, length of stay, previous stays, insurance type, family history of mental disorders
Experience with return to home care	x	– (only upon readmission^a^)	Challenges, resources, needs
Survey panel	x	x	(see below)
Youth outcomes			Healthcare outcomes, internalizing problems, externalizing problems, health-related quality of life
Caregiver well-being			Caregiver mental health, caregiver physical health, caregiver recovery, caregiver burden
Family processes			Family functioning, interparental relationship quality, parental dyadic coping, parent-child relationship, parent-youth dyadic coping, criticism

The qualitative interview is not included here, as it is conducted independent from the panel structure.

a Items related to inpatient stay are repeated in the case of any subsequent readmissions at a later time point.

#### 3.5.1 Sociodemographic information

At baseline, participants are asked to provide person- and family-related sociodemographic information using a total of 18 items. These include age, gender, country of residence, country of birth, education, vocational status, household income, financial stress, number of children, household size, migration background, primary language, and caregiving arrangement. A small number of sociodemographic variables may be subject to change over the course of the study period and are therefore repeatedly assessed at follow-up time points. These include country of residence, vocational status, financial stress, and caregiving arrangement.

#### 3.5.2 Clinical and treatment characteristics

At baseline, a total of six items assess the date of discharge, length of stay, number of previous inpatient stays, insurance type, reasons for inpatient treatment (parent-reported, based on ICD-10 categorization), and known mental disorders of other family members (self, other parent, sibling, other relative). In the event of any subsequent readmission at a later time point, caregivers will be asked to provide the date of admission and date of discharge or, optionally, duration as well as reasons for each inpatient stay episode since the last time point.

#### 3.5.3 Experience with return to home care

At baseline and in the event of any subsequent readmission at a later time point, caregivers are asked to provide free-text answers to three questions related to challenges, resources, and needs around their child’s return to home care (see study materials at OSF for specific items).

#### 3.5.4 Survey panel

##### 3.5.4.1 Healthcare outcomes (primary outcome)

Caregivers report on several healthcare outcomes following their child's discharge. These include follow-up patient-focused support (outpatient psychotherapy or psychiatric sessions, medication, aftercare sessions, social pedagogical support, self-help groups, and inpatient readmission) and family-focused support (case coordination, family-focused aftercare, youth welfare, school support, court support, family therapy, parenting counseling, peer groups, social support) since discharge (T0) or the last assessment point (T1, T2). If caregivers report any subsequent readmissions (since the previous time point), they are further asked to specify the date(s) and duration as well as reasons for the inpatient stays.

##### 3.5.4.2 Youth mental health (primary outcome)

Youth's internalizing and externalizing problems are assessed through caregiver-report using the internalizing and externalizing subscales of the short form of the Pediatric Symptom Checklist (PSC) (47). Caregivers report how often their child has experienced a total of twelve problem descriptions (five internalizing: e.g., “Worries a lot”; seven externalizing: e.g., “Has fights with other children”) on a 3-point scale from 0 (*never*) to 2 (*often*). The PSC has been widely used in studies with English-speaking participants and demonstrated excellent psychometric properties (48). The German version used in this study is an adapted short-form version of the German validation by Thun-Hohenstein and Herzog (49). It was developed using bilingual translation and back translation and is currently undergoing validation [for registration see (50, 51)]. For the present study, we adjusted the original six-month timeframe to one month in order to avoid overlaps for repeated assessments.

##### 3.5.4.3 Youth health-related quality of life (secondary outcome)

To assess children's quality of life within several domains (self-esteem, school, and peer relationships), we use the revised Health-Related Quality of Life Questionnaire (KINDL-R) (52). For each domain, caregivers rate four items on how their child has fared in the past week (e.g., “was proud of themselves”) on a 5-point scale from 1 (*very rarely*) to 5 (*very often*). The KINDL-R is applicable to the full age range of children and adolescents and has been shown to correlate moderately with child-report (53). Multiple studies have concluded its reliability and validity (53, 54).

##### 3.5.4.4 Caregiver health

Caregivers’ own mental and physical health problems are assessed with a German version of the Short-Form Health Survey (SF-12) (55, 56). The questionnaire measures physical and mental health-related well-being. Respondents evaluate the impact of health problems on their everyday life on a total of six items for physical and mental health, respectively. We use all six mental health items (e.g., “How often have you been restricted in your private or professional activities due to mental or emotional problems?”) and the general physical health item (i.e., “How is your physical state of health in general?”). The Short-Form Health Survey has been widely used internationally and extensively evaluated across different versions (56). The mental health scale of the present German version has demonstrated high reliability (55).

##### 3.5.4.5 Caregiver recovery

The Recovery Questionnaire – Parent Report (ReQuest-P) (57) assesses recovery-related processes from the perspective of caregivers. Respondents are prompted to evaluate 22 statements regarding their experience of recovery in relation to their child's mental health problems (e.g., “I know there are different ways in which I can help my child”) on a four-point scale from 0 (*not at all*) to 3 (*completely*). The measure was specifically developed for use within the context of youth mental healthcare based on theoretical considerations and interviews involving youth with mental health problems and their parents. Validation within an independent sample suggested good internal consistency and acceptance (57). The ReQuest-P is currently only available in its original English version; thus, we translated the original items of this measure into German for the purpose of this study using a forward-backward translation procedure. Two native German-speaking members of the research team, one of whom had additional training in linguistics, independently produced forward translations of the original items. Discrepancies between the two versions were resolved by consensus, with sematic equivalence as the primary criterion. The finalized German version was subsequently back-translated into English using DeepL, with the output reviewed by a team member naïve to the original instrument. Comparison of the back-translation with the original indicated high semantic correspondence across items, with no substantive deviations.

##### 3.5.4.6 Caregiver burden

The German Caregiver Strain Questionnaire – Short Form (CGSQ-SF11) will be used to assess caregiver burden (58, 59). The eleven items consist of questions about the impact of a child's problems (e.g., “Do your child's problems interrupt your personal time?”) rated on a 5-point Likert scale ranging from 1 (*not at all*) to 5 (*very much*). Validation studies have demonstrated construct validity for an overall strain score as well as three subscales, namely objective strain, internalized subjective strain, and externalized subjective strain. The instrument has further proven adequately reliable both in terms of internal consistency and test-retest correlations (58, 59).

##### 3.5.4.7 Family functioning

We employ the *General Family Functioning* subscale of the Family Assessment Device (FAD-GF) in its German validated version (60). Respondents rate how twelve items describing family functioning (e.g., “We can support each other in a crisis”) apply to their family on a 5-point scale from 1 (*very rarely*) to 5 (*very often*). The FAD is based on the McMaster model of family functioning (61) and has been widely used across various translations (62). Both the original version as well as the German translation have proven to be psychometrically sound (60, 63).

##### 3.5.4.8 Interparental relationship quality

Caregivers who are currently in a relationship will complete the four-item Couples Satisfaction Index (CSI-4) in its German translation, which was developed from expert comparisons between two separate translations and native-speaker back-translations and is currently being validated (64). Respondents are asked to rate the overall degree of happiness within their relationship on a 7-point scale from 0 (extremely *unhappy*) to 6 (*perfect*) and further evaluate three statements about their relationship (e.g., “I have a warm and comfortable relationship with my partner”) on a 6-point scale ranging from 0 (*not at all [true]*) to 5 (*completely [true]*). The original English version is widely used and has shown good internal consistency, validity, and measurement accuracy (65).

##### 3.5.4.9 Parental dyadic coping

Caregivers who state that they are currently in a couple relationship will further complete the short-form version of the Dyadic Coping Inventory (DCI-K) (66). The questionnaire consists of 12 items describing how couples deal with stress together (e.g., “We try to tackle the problem together and look for concrete solutions”). Respondents rate how often these statements apply to their relationship on a scale from 1 (*very rarely*) to 5 (*very often*). The DCI has demonstrated good psychometric properties in its original as well as its short-form version (67, 68).

##### 3.5.4.10 Parent-child relationship

The parent-child relationship is assessed using the German Multiperspective Parent-Child Relationship Questionnaire (M-PCR) (69). Caregivers rate their agreement with 14 statements (seven items per subscale) about their relationship with the child or adolescent (e.g., “My child and I tend to ignore each other”) on a 5-point scale ranging from 1 (*do not agree at all*) to 5 (*totally agree*). The questionnaire has been developed in samples of parents with preschool children, where it has been shown to be reliable and sensitive (69), but since then it has also been used in studies with parents of children up to adolescent age (70).

##### 3.5.4.11 Parent-youth dyadic coping

Parental responses to youth stress will be assessed using the Parent-Youth Dyadic Coping Inventory (PY-DCI) (66). Using a total of 13 items, caregivers rate how they usually respond when their child expresses stress (e.g., “I make them feel understood”) on a 5-point scale from 1 (*very rarely*) to 5 (*very often*). Subscales include problem-oriented, emotion-oriented, and negative parent-youth dyadic coping. This measure has been established in a number of previous studies with different samples (71–73).

##### 3.5.4.12 Parental criticism

We assess the criticism dimension of the Expressed Emotion construct using the one-item Perceived Criticism Measure (PCM) (74), which asks caregivers to rate the question “How critical have you been of him/her?” on a 10-point scale from 1 (*not at all*) to 10 (*very*). A German version of the PCM has been established, showing shared variance with more comprehensive measures of Expressed Emotion and moderate agreement between ratings of relatives and patients (75, 76).

#### 3.5.5 Interview

Online interviews follow a semi-structured format (see study materials at OSF for the interview manual). The interview guide was developed within the research team in accordance with guidelines for qualitative interview construction (77) and is structured to explore caregivers’ experience with post-discharge care, specifically focusing on the challenges and needs around post-discharge care as well as coping resources and support systems. Interviews are expected to last between 45 and 60 min.

### 3.6 Data analysis

The main RQs will be examined in multiple analyses outlined in the following. Analysis approaches were chosen based on power, design, and data considerations. The family processes examined in this study and their influence on the study outcomes are assumed to change over the post-discharge period. For example, caregiver burden has been suggested to be differentially associated with readmission depending on the time of assessment (8, 35). This requires models that account for the time-varying nature of predictors. Moreover, research has begun to differentiate between single and repeated readmission risks [e.g., (78)], as frequent readmissions may represent specific risks that need to be identified. Accounting for recurrent hospitalizations not only addresses this issue but also leverages the full extent of available information. Therefore, to examine RQ 1a, inpatient readmissions will be modeled using generalized cox proportional hazard models extended to model repeated occurrence (79). Caregiver burden will be examined as the primary (confirmatory) predictor under study in all analyses, with additional family variables tested in exploratory analyses. RQ 1b will investigate the longitudinal development of youth mental health problems as outcome. The related hypotheses will be analyzed using Latent Growth Curve Modeling (LGCM), which is particularly suitable to model recovery trajectories over time (80). The model allows for including both time-invariant (e.g., gender, socioeconomic status) and time-varying predictors (e.g., family processes). The reciprocal relationships hypothesized for RQs 2 and 3 will be tested using a Cross-Lagged Panel Model (CLPM), which is well-suited for examining direct reciprocal associations between two variables across repeated assessments while accounting for autocorrelation (81). The model has previously been applied to model associations between youth problems and caregiver strain (36). To test the proposed mediation through parent-child interaction, we will examine criticism as the best-studied interaction variable. If the sample size and model stability permit, analysis will further include a random intercept component to differentiate stable between-family differences from dynamic within-family change (see (82) for specifications of the Random Intercept Cross-Lagged Panel Model [RI-CLPM])). Given the broad age range of youth in our study, age will be included as a covariate in all models. RQ 4 is addressed by synthesizing quantitative and descriptive data on caregivers’ subjective distress and follow-up support together with qualitative data from interview transcripts (see section 3.5.5) and open-field answers (see section 3.5.3). Qualitative data will be analyzed using thematic analysis (83), a qualitative method aimed at extracting themes that are central to the topic under study. Deductively predefined themes will be expanded inductively within the research team. Using a coding reliability approach (84, 85), codes will be examined for consistency when applied to a random subset of the material. Reliability estimates will be used to inform iterative refinement of code definitions in conjunction with discussion within the research team. Code development will be considered complete when codes demonstrate both satisfactory reliability (*k* ≥ .80) and conceptual clarity. Disagreements will be resolved through consensus in the research team. Synthesis will follow complementary triangulation. Quantitative and qualitative data will be analyzed separately, then integrated to explore how the data provide complementary insights into caregiver experiences. Linkage at the individual level will enable comparisons across caregivers differing in burden, readmission history, and clinical presentation of their child. Mixed-methods analyses will use all available data regardless of follow-up attrition.

#### 3.6.1 Sample size and power considerations

Studies with difficult-to-reach and highly strained populations must find a reasonable compromise between statistical precision and practical feasibility. The achievable sample size is expected to be small and likely to contain some missing data. A precise power calculation for the models described above would require extensive simulations that exceed feasibility. Instead, we refer to statistical recommendations, empirical references, and practical considerations in determining our target sample size.

Empirically, previous studies suggest small (prospective) to large (correlational) effect sizes for the proposed associations (36), and have detected significant associations of family variables with post-discharge outcomes, with sample sizes as low as 40 to 50 (23, 86, 87). The current target sample size of *N* = 70 is set to exceed these benchmarks while taking them as a reference for realistic recruitment prospects.

Statistically, repeated assessments will further yield higher data count, and thus, higher statistical power. Specifically, for proportional hazards regression analysis (RQ 1a), simulation-based recommendations set a threshold of 10 event occurrences per predictor variable. With an expected readmission rate of up to 30%, corresponding to 21 expected events, a sample size of *N* = 70 meets these requirements. The approach developed by Andersen and Gill (79) further enhances statistical power by pooling events. Of note, the model assumes independence of events, an assumption that is contested by evidence for higher hospitalization risk given previous readmissions (1). Approaches that stratify subsequent readmission [e.g., Prentice-Williams-Peterson models, see (88)] were discarded given the small expected number of higher-order events. To still account for within-family correlations, robust standard errors will be estimated and the number of readmissions will be included as a time-dependent covariate, as suggested by Castañeda and Gerritse (89).

We will report sample sizes and missingness patterns across waves and conduct attrition analyses comparing completers and noncompleters in terms of baseline characteristics. Analyses will assume MAR (conditional on observed demographic and clinical variables). If associations are found, sensitivity analyses will be conducted including the relevant variables as auxiliary variables. Both LGCM (RQ 1b) and (RI-)CLPM (RQs 2 and 3) effectively handle missing data through Full Information Maximum Likelihood (FIML) estimation. Specifically, estimates will be based on Maximum Likelihood with Robust Standard Errors (MLR), a method shown to provide stable and unbiased estimates in small (*N* < 100) and incomplete samples (90). Should recruitment fall short of the target sample, analyses will be adapted to maintain statistical validity by focusing on primary hypotheses and simplifying multivariable models as needed. As a contingency, simplified mixed-effects growth models will be estimated if LGCM do not converge, reducing model complexity by treating growth parameters as observed random effects rather than latent variables. This approach is less susceptible to non-convergence under small-sample conditions while preserving the core growth hypothesis. For CLPM, model constraints will be applied to improve estimator stability in case of non-convergence, namely setting residual variances, autoregressive and cross-lagged paths as equal across timepoints.

### 3.7 Data management

Data management procedures comply with the European General Data Protection Regulation (GDPR) and were approved by the institutional ethics committee (see section 5). All data is collected and stored on ISO27001 certified user- and password-protected university servers with exclusive access authorization by project members.

Data is anonymized one month after the end of data collection by deleting personal information used for project administration and stored indefinitely on university servers. Participants can request access to and deletion of their data up to this point. Anonymized data will be shared with the scientific community via OSF according to open science principles after reviewing and removing any variables that may compromise the anonymity of participants.

Interviews are recorded using Zoom licenses tailored to meet high security and data protection requirements. Recordings are accessed via a cloud server located in Germany, transferred to university servers, and deleted on the cloud server immediately after the file transfer. Recording files are only accessible to project members and processed on-site. The recording method produces video data that is deleted immediately after isolating the audio tracks. Audio files are deleted after one year or once data coding is complete. Until then, files are stored using a recording code that is separate from the participant pseudonym. Transcripts of recordings will be reviewed for any mention of identifying information (name, locations, date of treatment), which is replaced by anonymous placeholders.

## 4 Discussion

This study is among the first to examine caregiver perspectives on youth recovery and reintegration after inpatient psychiatric treatment within European healthcare contexts. Data from this research project will highlight the burden and needs of caregivers around their child's return to home care. Results will expand on the limited evidence on caregiver and family factors for youth mental health post-discharge. In addition to prognostic research questions, the multinational sample of German-speaking countries is a novelty of this study that allows for capturing the current state of follow-up care and family support in these regions.

The study addresses several gaps in the field by using standardized measures to prospectively examine caregiver factors in European healthcare contexts. A methodological strength of the study is the use of an analytical approach that models recurrent events. As a recent review has highlighted, studies on readmission outcomes have yet to adopt new methodological advances in modeling approaches (91). Similarly, the use of models that include time-varying predictors across analysis approaches leverages the repeated-measures data and accounts for the known time sensitivity of family processes. The mixed-methods design further addresses the highlighted benefit of including qualitative data in research on caregiver burden (5). Synthesis of data from different methodological approaches may identify aspects of the caregiver experience that might otherwise be missed.

### 4.1 Limitations

The present study has limitations that warrant mention. First, recruitment will target a highly burdened target population. Consequently, enrollment and retention rates are expected to be low. The target sample size was set to account for these anticipated difficulties. Furthermore, missing or incomplete data in this prospective study is expected to be higher than in general populations as a result of the increased burden. To minimize attrition over the course of the study, short forms of questionnaires are used where possible, and financial compensation is set up to incentivize continuous participation. Furthermore, analytical approaches were chosen to best account for the potential data limitations. The target sample size set for this study is nevertheless limited and must be understood as providing probing results rather than definitive evidence. Second, the study period of six months makes it impossible to examine longer-term developments. Effects of family-level factors and interventions are often more pronounced over longer timeframes (92). Nevertheless, the study timeframe captures a critical period, as most readmissions occur within the first three months (1). The results of the three assessments (shortly after discharge and after three months and six months) will provide valuable insights into the immediate and medium-term post-discharge period but will not capture longer-term outcomes beyond this timeframe. Third, all measurements rely on caregiver report, and several instruments are administered in short-form versions that are still undergoing validation. These short forms were deliberately selected to reduce participant burden. However, this choice entails reduced certainty regarding reliability and measurement precision compared with fully validated instruments. Although the original versions have demonstrated good psychometric properties, the performance of the abbreviated forms in this population may affect the robustness of the findings. Further, issues around disagreements and inaccuracies of caregiver proxy-reports on youth mental health outcomes compared to direct youth self-reports have been highlighted (93, 94). The present study focuses exclusively on caregiver perspectives and thus relies on their report on youth outcomes, which must be taken into account when interpreting the findings. This design was chosen for its feasibility and the explicit focus of the study, given that caregivers’ perceptions of youth problems are particularly meaningful in the context of caregiver burden and youth service use (5). However, any inferences regarding youth mental health will be restricted to caregiver perceptions and may differ from conclusions based on youth self-report. In addition, the inclusion of only one caregiver means that the inherently dyadic family processes examined in this study lack the perspective of additional caregivers in the family. Relevant congruencies or discrepancies between family members may thus be missed. Fourth, the observational nature of the study bears the limitation that received support services will neither be controlled nor randomized and will thus be confounded by a combination of objective and perceived need as well as the availability and access to services. This inherent shortcoming means that we cannot infer causal effects of follow-up support services. Last, the study is focused on family processes and therefore does not encompass all relevant determinants of post-discharge youth outcomes. While we will adjust for key prognostic indicators (i.e., symptom severity, previous hospitalizations), other relevant determinants (such as treatment and medication adherence, diagnostic characteristics, or patients’ health beliefs) are not modeled. This reflects the analytic focus on family processes rather than an attempt to comprehensively model outcomes. Of note, the broad age range of youth may introduce age-related variability in clinical presentation and prognosis, treatment and school involvement, as well as in the examined family processes. To address this, models will be adjusted by including age as a covariate.

Despite these limitations, the study design uniquely allows for examining temporal dependencies of caregiver factors and youth mental health in the immediate post-discharge period. Insights into the role of caregiver and family factors may help identify targets for improving family support during and after the transition out of hospital care. For example, the view of putting caregiver burden at the core of recovery-promoting family processes highlights coping resources as potentially crucial protective factors. Qualitative data will further elucidate which resources caregivers find particularly helpful or lacking.

## 5 Ethics and dissemination

A protocol of this study (including all procedures, measures, and materials) was reviewed and approved by the institutional ethics committee of the University of Vienna (reference number: 01291; date of approval: February 2, 2025). Members of the target population were not involved in the development of this protocol. The full text of informed consent forms is available on OSF. Before consenting to participation, interested caregivers will be comprehensively informed about the study procedures and their participant rights. The study procedures are not expected to pose risks to the mental or physical integrity of participants. Participants may experience discomfort during surveys or interviews. They are informed in advance that they can skip, postpone, or discontinue study participation at any point and are encouraged to contact study staff. Such instances are documented by study staff, including the concern and the action taken. In addition, outpatient and inpatient services as well as emergency telephone numbers are listed as crisis support services.

Findings from this study will be disseminated in international peer-reviewed journals. The resulting publications will be posted as preprints in the OSF PsyArXiv repository unless the policies of the respective publisher restrict or prohibit it.
